# Application of a New Carbon Black Filler in SBR Composites

**DOI:** 10.3390/polym17030358

**Published:** 2025-01-28

**Authors:** Junan Zhou, Bainan Tang, Liangchen Yu, Junping Song, Zepeng Wang

**Affiliations:** 1 College of Mechanical and Electrical Engineering, Qingdao University of Science and Technology, Qingdao 266061, China; 18266629639@163.com (J.Z.); t17661241685@163.com (B.T.); y1047236506@163.com (L.Y.); 2 Sino-German Institute of Science and Technology, Qingdao University of Science and Technology, Qingdao 266061, China; 02165@qust.edu.cn

**Keywords:** SBR, new carbon black, N660 carbon black, reinforcement materials

## Abstract

The microstructure and properties of a new type of carbon black produced by a domestic company through a new process were systematically characterized by scanning electron microscopy (SEM), energy dispersive spectroscopy (EDS), Raman spectroscopy, and X-ray diffraction (XRD). The vulcanization properties, mechanical properties, and electrical conductivity of the new carbon black with different filler amounts were investigated in styrene butadiene rubber (SBR), using the traditional reinforcing filler N660 carbon black as a control. The experimental results demonstrate that the new carbon black exhibits a stratified structure with a specific surface area of 345.96 m^2^/g, and its particle size distribution is primarily concentrated within the 0.1–1 μm range. When the filling ratio was 30 phr/100 phr, the tensile strength of SBR composites filled with the new carbon black increased by 12.3% and the tear strength increased by 9.6% compared with those filled with N660 carbon black. In summary, the new carbon black can significantly improve the comprehensive performance of SBR composites and reduce the production cost. This provides a new type of material for the rubber industry that takes into account both economy and performance while also providing reference data for basic research in the field of SBR.

## 1. Introduction

Carbon black is mainly composed of carbon with small amounts of nitrogen, hydrogen, and oxygen and has an amorphous carbon structure [1]. With its excellent reinforcing ability, carbon black is widely used in industrial production [2]. However, the preparation process of traditional carbon black has high energy consumption and serious pollution emission, so the development of high-performance rubber-reinforcing materials with low cost and low pollution characteristics has become a research focus of academic and industrial attention [3]. SBR is a general-purpose synthetic rubber made from butadiene and styrene through polymerization, which plays an irreplaceable role in various fields due to its excellent electrical conductivity, tear resistance, environmental friendliness, and low cost. However, pure SBR itself has low strength and modulus, and in practical applications, it is often necessary to enhance its comprehensive performance by adding reinforcing materials [4].

Chengyu Tang et al. [5] found that the new homemade high-structure carbon black with high specific surface area and high structural characteristics can significantly improve the tensile and tear strength of natural rubber while optimizing the vulcanization characteristics and showing excellent reinforcing ability. Lili et al. [6] explored the synthesis of graphene oxides and reduced graphene by using graphite foil waste as a carbon precursor, which provided a new way for the preparation of low-cost graphene materials. Yanchen Fang et al. [7] used plasma-modified pyrolytic carbon black and filled it into natural rubber, and found that the modified carbon black had smaller particle size and more uniform distribution and bonded more tightly with the rubber matrix. They also found that the fatigue life of the composite material was extended by 107%. Lei Gao et al. [8] showed that lignin-reinforced emulsion-polymerized SBR could form a good interfacial bond and significantly improve material properties. Tilun et al. [9] improved the filler dispersion and mechanical properties of SBR by using a waste catalyst as filler and utilizing its micro porous structure and active components.

A new carbon black prepared by a domestic company using a new process, while reducing the emission of pollutants in the production process, retains and improves the reinforcing properties of carbon black, and the cost is only two-thirds of the traditional N660 carbon black. As a classic medium reinforcing material, N660 carbon black is widely used in industrial fields [10]. Compared with the traditional high-reinforcing carbon black, the comparative study of N660 carbon black and the new carbon black can better reflect the potential advantages of the new carbon black. By systematically analyzing the effect of the new carbon black produced by the new process on the performance of SBR, this study provides important theoretical support and practical reference for the new carbon black to partially replace the N660 carbon black as a rubber-reinforcing material.

## 2. Materials and Methods

### 2.1. Experimental Raw Materials and Formulations

The raw materials and formulations (mass/phr) for the SBR experiments were as follows: SBR1502 100, zinc oxide (ZnO) 5, stearic acid (SA) 1, Si 69 2, accelerator CZ 1.5, rubber accelerator TMTD 1.5, sulphur 0.5, N660 carbon black, and new carbon black (variable).

### 2.2. Instruments and Equipment

BL-6175-BL double-roller opener (Baolun Precision Testing Instrument Co., Ltd., Dongguan, China); GT-7016-AR pneumatic automatic slicer (LX-A Shore’s Rubber Hardness Tester (Shanghai Liuling Instrument Factory, Shanghai, China); MDR2000 Rotorless Vulcanizing Instrument (Alpha Corporation, Dulles, VA, USA); HS-100T-2 Rubber Vulcanizing Machine (Shenzhen Jiaxin Electronics Co., Ltd., Shenzhen, China); GT-AI-7000M Tensile Tester (High Speed Rail Technology Co., Ltd., Beijing, China); ZGS-170808T-722 Wide-Frequency Dielectric Impedance Spectrometer (Beijing Huidexin Technology Co., Ltd., Beijing, China).

### 2.3. Experimental Scheme

SBR composites and specimens were prepared to study the effect of new carbon black on SBR vulcanization characteristics, mechanical properties, and electrical conductivity. The specific experimental steps are as follows:

Before beginning, clean the rollers of the mixing machine. Next, weigh the SBR and place it into the mixer to soften it until the rubber surface becomes smooth and uniform, followed by mixing. Gradually add the temperature-sensitive raw materials, ensuring they are well mixed, then add the reinforcing materials. Finally, add sulfur, mix thoroughly, adjust the roller pitch, and perform the triangular package operation 8 times until the rubber is in sheet form and set aside. After letting the rubber rest for 24 h, measure the vulcanization curve using a rotorless vulcanizer to determine the optimal vulcanization time (t_90_). Then, carry out vulcanization at 160 °C for (t_90_ + 2 min) under a pressure of 10 MPa and prepare standard samples. Test the prepared SBR composites and analyze them for vulcanization properties, mechanical properties, and electrical conductivity.

### 2.4. Performance Test

A scanning electron microscope (SEM) was utilized to observe the microscopic morphology of the new carbon black as well as the cross-section structure of the rubber composite with a resolution of 1 nm (accelerating voltage 15 kV). The vulcanization properties were tested according to the GB/T 9869-2014 standard, the tensile properties were tested according to the GB/T 528-2009 standard, and the tear strength was tested according to the GB/T 529-2008 standard. A broadband dielectric impedance spectrometer was used to determine the conductivity of the composites. The specific surface area of the new carbon black was tested using the Brunauer–Emmett–Teller method (BET). Graphitization degree and structural defects of new carbon black were analyzed by Raman spectroscopy at a fixed collection angle of 90° and by the ratio of the intensity of the D peak to that of the G peak (ID/IG). X-ray diffraction (XRD) was used to analyze the physical phase of the new carbon black, and the compacted samples were placed on blank slides and sent to the sample chamber, using Cu target Kα-rays with a scanning range of 0–90°. Differential scanning calorimetry (DSC) was used to characterize the glass transition temperature (T_g_) of the composites.

## 3. Results and Analysis

### 3.1. Specific Surface Area and SEM

**Figure 1:**
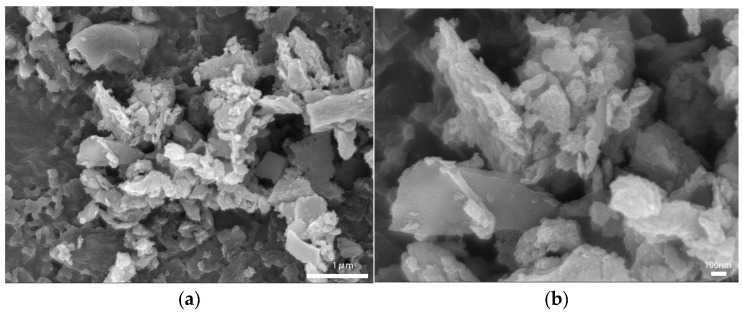
SEM of new carbon black: (**a**) magnification 20,000 times; (**b**) magnification 50,000 times.

BET test results show that the specific surface area of the new carbon black is 345.96 m^2^/g. Using SEM at 20,000 times magnification to visualize the new carbon black material, as shown in Figure 1a, it can be seen that the new carbon black has a stratified structure and is irregular in shape. The stratified structures of different sizes are closely packed together to form carbon black aggregates, and there is a certain amount of void between the aggregates, contributing to the overall roughness of the structure. Using SEM at 50,000 times magnification to visualize the new carbon black material, as shown in Figure 1b, it can be seen that the new carbon black is mainly in the form of flakes or lumps, with a rough surface and irregular edges, and the voids between particles are more obvious and have a larger aspect ratio.

### 3.2. Laser Particle Size

**Figure 2:**
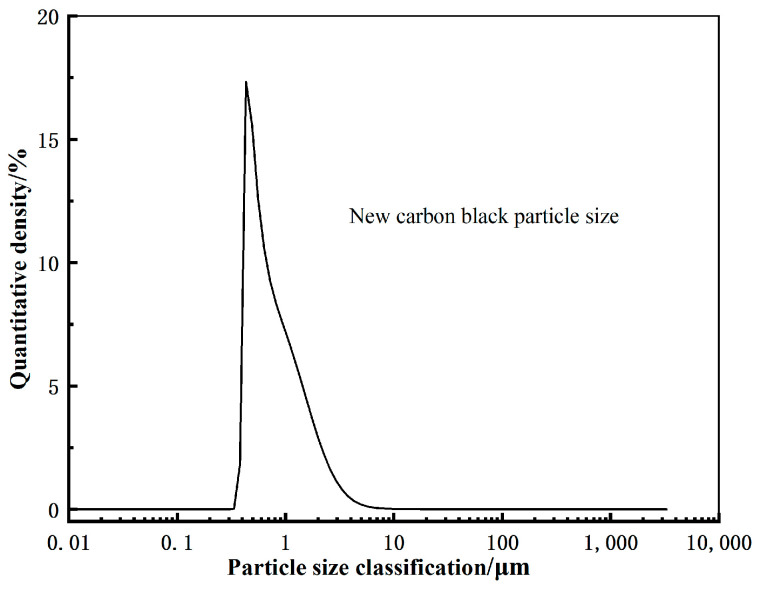
Particle size of new carbon black.

Figure 2 shows that the particle size of the new carbon black is more concentrated, and its peak corresponds to a smaller particle size, mainly in the range of 0.1–1 μm, with micrometer and nanometer fine particle characteristics, showing good dispersion and filling uniformity. This particle size distribution helps the new carbon black form a uniform dispersion network within the rubber matrix, preventing the issue of stress concentration caused by particle aggregation [11].

### 3.3. EDS

**Figure 3:**
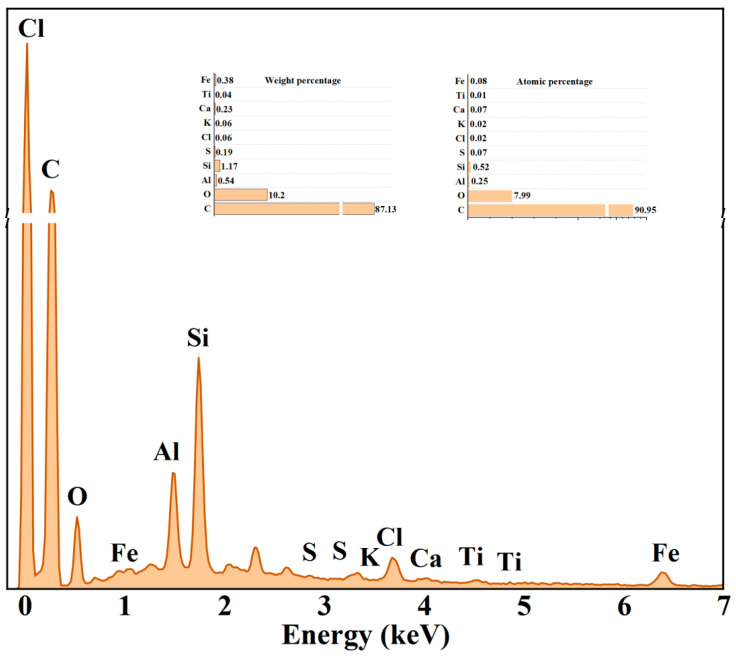
EDS of the new carbon black.

As shown in Figure 3, through energy dispersive spectrometer (EDS) analysis of the new type of carbon black, the carbon mass fraction is 87.13%, and the atomic fraction is 90.95%; the oxygen mass fraction is 10.2%, and the atomic fraction is 7.99%. The presence of oxygen imparts a certain degree of oxidizing properties to the surface of the carbon black particles, which facilitates chemical reactions with the rubber matrix and enhances interfacial bonding. Additionally, the presence of impurities such as aluminum, silicon, and calcium provides the potential for performance improvement of the composite material. These impurities create a synergistic effect with the reinforcing role of carbon black, making it a multifunctional and cost-effective rubber filler.

### 3.4. Raman Spectra

The D peak and G peak in Raman spectroscopy are key peaks used to characterize the properties of carbon materials. The D peak is closely related to the defects and amorphous carbon in the material, reflecting the degree of disorder, while the G peak is associated with the C-C stretching vibration in graphite crystals, reflecting the degree of graphitization or structural order of the material [12].

**Figure 4:**
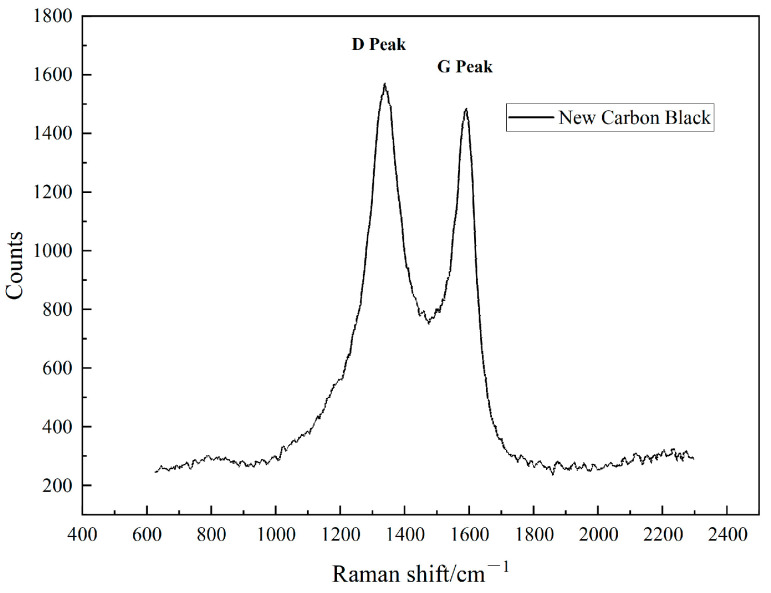
Raman spectra of the new carbon black.

As shown in the Raman spectra in Figure 4, the D peak of the new carbon black appears at 1570 cm^−1^, and the G peak is located at 1485 cm^−1^. Both peaks are pronounced, with the ID/IG ratio greater than 1, suggesting a higher content of disordered or defective carbon, along with some degree of graphitization. The high intensity of the D peak suggests a greater presence of defective structures and amorphous carbon, while the moderate intensity of the G peak indicates that the new carbon black possesses both a disordered structure and the ability to form a conductive network while still maintaining some level of graphitization. Increased structural disorder and defects in the new carbon black enhance the number of active sites on its surface, providing more opportunities for bonding with the rubber matrix or other components. This can strengthen the filler–matrix interactions and, potentially, improve the properties of the composites.

### 3.5. XRD

**Figure 5:**
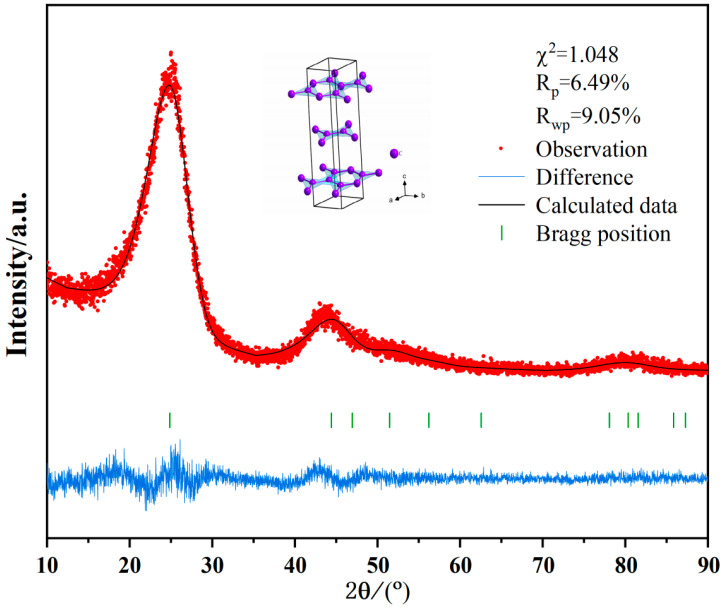
XRD refinement of the new carbon black.

Figure 5 shows the XRD refinement of the new carbon black. The cell parameters were calculated by performing Rietveld refinement on the XRD patterns using Highscore plus (ver4.8) software with an initial refinement model of C (space group of R-3m, ICDD = 199,068). The back bottom was fitted using the polynomial function and the peak profile was fitted using the pseudo-Voigt function. Refinement confidence criteria references: R_wp_ < 10%, R_p_ < 10% and the goodness of fit (χ^2^) < 2. The black computed spectral lines in the refined plots have a better fit to the red measured curves, indicating the high reliability of the refined results, which is further supported by the lower confidence factors (R_wp_, R_p_, and χ^2^) [13].

From the match between the Bragg position and the diffraction peaks, it can be seen that the new carbon black exhibits strong crystalline features with a certain degree of long-range ordering within its crystal structure. The main diffraction peaks are concentrated between 2θ = 20°~30°, with cell parameters a = b = 2.38416, c = 10.60064, suggesting that the crystals may have hexagonal symmetry. The lattice angles are α = β = 90°, γ = 120°, the cell volume = 52.18346^3^, and the diffraction peak width is moderate, indicating that the grain size of the new carbon black is small, with the potential for microscopic strain on its surface.

## 4. Discussion of Results

### 4.1. Dispersibility

Good dispersibility ensures that stress is evenly transferred within the rubber material under external forces, preventing stress concentration and improving mechanical performance [14].

**Figure 6:**
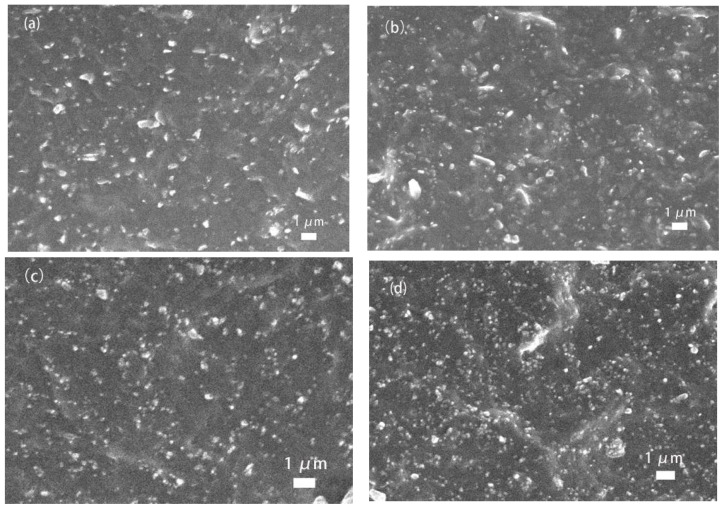
SEM images of different amounts of new carbon black- and N660 carbon black-filled SBR: (**a**) filled with 20 phr new carbon black; (**b**) filled with 40 phr new carbon black; (**c**) filled with 20 phr N660 carbon black; (**d**) filled with 40 phr N660 carbon black.

After the NBR composites were subjected to low-temperature embrittlement, the dispersion of the new carbon black particles in the rubber matrix was observed by SEM. As shown in Figure 6a, in the 20 phr new carbon black-filled SBR composites, the carbon black particles were uniformly distributed with moderate particle spacing, indicating good dispersion and some reinforcing effect. Despite the irregular shape and large size of the new carbon black particles, the overall interfacial bonding was good. As shown in Figure 6b, when the filling amount of the new carbon black was increased to 40 phr, the particle density increased significantly, and significant agglomeration also occurred. The bright areas show larger agglomerates, which may lead to localized stress concentration. As shown in Figure 6c, in the 20 phr N660 carbon black-filled SBR composites, the N660 carbon black particles were more uniformly distributed, with finer and denser distribution of bright spot areas. As shown in Figure 6d, when the filling amount of N660 carbon black was increased to 40 phr, although the particle density was increased and some agglomeration phenomenon appeared, the size of the agglomerates was smaller, and the overall dispersion was better than that of the new carbon black.

From the above analysis, it can be seen that both the new carbon black and N660 carbon black exhibit strong molecular chain adsorption and entanglement in SBR.

### 4.2. DSC

**Figure 7:**
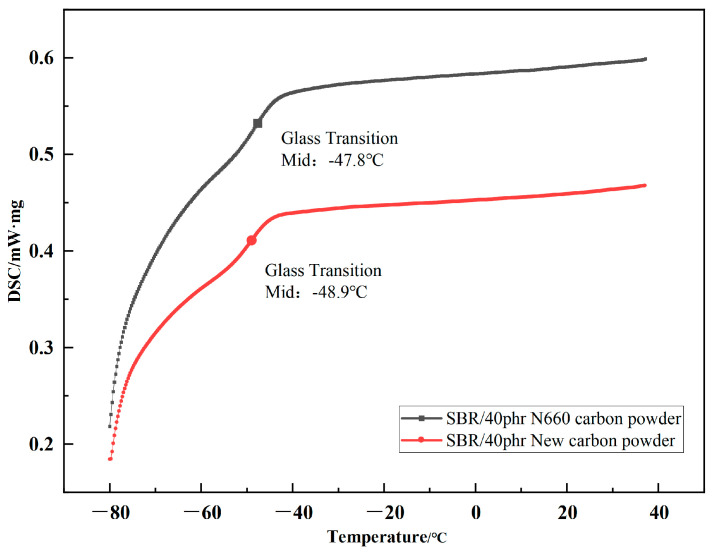
The glass transition temperature of 40 phr N660 carbon black- and new carbon black-filled SBR.

As shown in Figure 7, the glass transition behavior of the SBR composites was investigated by DSC. It was found that the median T_g_ of the new carbon black-filled SBR was −48.9 °C, which is slightly lower than that of the conventional N660 carbon black-filled SBR (T_g_ = −47.8 °C) by about 1.1 °C. This suggests that the new carbon black exerts a weaker restriction on the movement of the rubber molecular chain segments.

This phenomenon is closely related to the microstructure of the new carbon black and its interfacial interaction with the matrix due to its high specific surface area and uniform particle distribution. The new carbon black can form a more homogeneous filler network in the matrix. This network helps reduce the excessive binding of the local chain segments, allowing the rubber molecular chains greater freedom of movement [15]. Additionally, the lower surface energy of the new carbon black may reduce the physical forces between it and the SBR molecules, thus lowering the stiffness of the composite. The lower T_g_ indicates that the material exhibits superior flexibility and cold resistance at low temperatures, providing a potential advantage for applications involving the new carbon black in low-temperature conditions.

### 4.3. Vulcanization Properties

The effects of the dosage of new carbon black and N660 carbon black on the vulcanization characteristics of SBR composites are shown in Table 1 and Table 2. The maximum torque (M_H_) of the SBR composites increased from 16.123 dN·m to 20.754 dN·m with the increase in the dosage of new carbon black, which reflects the shear modulus of the vulcanized rubber and represents the strength of interaction between the rubber and the filler. This change indicates that the incorporation of the new carbon black significantly increased the cross-link density of the SBR composites. Meanwhile, the minimum torque (M_L_) increased from 1.057 dN·m to 2.122 dN·m, indicating that the mobility of the composites gradually weakened with the increase in the content of the new carbon black. In addition, the difference between the maximum and minimum torque (M_H_ − M_L_) increased from 15.066 dN·m to 18.628 dN·m, which is an important indicator of the degree of vulcanization cross-linking [16]. Compared with the S1-S4 samples (filled with N660 carbon black) and the S5-S8 samples (filled with new carbon black), the degree of cross-linking between the two samples was similar at 20–30 phr, and with the further increase in filler, the dispersion of the new carbon black was slightly worse than that of N660 carbon black, resulting in a slightly lower degree of cross-linking in SBR composites filled with new carbon black than N660 carbon black.

The small change in t_90_ indicates that the addition of the new carbon black has a limited effect on vulcanization efficiency, allowing for the maintenance of high vulcanization efficiency. The vulcanization time of the S5–S8 samples ranges from 6:05 min to 6:17 min, which is significantly shorter compared to the S1–S2 formulations using N660 carbon black. This demonstrates that the new carbon black has a beneficial effect on promoting the vulcanization reaction. This improvement is primarily due to the active functional groups on the surface of the new carbon black. As a result, the cross-linking reaction is accelerated, making the vulcanization system more efficient.

### 4.4. Mechanical Properties

The effect of the dosage of new carbon black and N660 carbon black on the mechanical properties of SBR composites is shown in Table 3. The tensile strength of SBR composites increased significantly with the increase in carbon black and new carbon black filler. At 20 phr, the tensile strength of the new carbon black-filled SBR was 10.4 MPa, which was 7.2% higher than that of the N660 carbon black-filled SBR of 9.7 MPa, and the two reinforcing materials had similar enhancement effects at low filler levels. When the filling amount increased to 30 phr, the tensile strength of the new carbon black-filled SBR increased significantly to 18.3 MPa, which was 12.3% higher than that of the N660 carbon black-filled SBR at 16.3 MPa, showing a better reinforcing effect. When the filling amount was increased to 40 phr, the tensile strength of the new carbon black-filled SBR was 22.2 MPa, which was 4.4% higher than that of the N660 carbon black-filled SBR at 21.27 MPa. When further increased to 50 phr, the tensile strength of the new carbon black-filled SBR was 23.6 MPa, which was 5.2% higher than that of the N660 carbon black-filled SBR of 22.43 MPa. It is true that the new carbon black is slightly less dispersed compared to N660 carbon black, but the higher tensile strength observed in the new carbon black-filled SBR composites can be attributed to its unique lamellar structure as well as a degree of oxidizing properties on its surface, which facilitates its chemical reaction with the rubber matrix and enhances the interaction between the carbon black particles and the SBR matrix. In summary, the tensile strength of the new carbon black-filled SBR was always higher than that of the N660 carbon black-filled SBR at the same filling amount, and the reinforcing effect was the most significant at 30 phr, but the two reinforcing effects were gradually close to each other with the increase in the filling amount.

The tear strength of SBR composites increased with the increase in both carbon black and new carbon black filler amount. Compared with N660 carbon black, the new carbon black showed more significant reinforcement effects at different filler levels. The tear strength of SBR filled with new carbon black was always higher than that of SBR filled with N660 carbon black under the same filling amount, and the tear strength of SBR filled with new carbon black reached 43.86 MPa at 30 phr, while that of SBR filled with N660 carbon black reached 40.02 MPa, with an increase in tear strength of 9.6%. The network structure of the molecular chains of the composites was enhanced with the increase in the filler amount, and the elongation at break increased significantly. However, when the filler amount exceeded 40 phr, the elongation at break tended to stabilize or even decrease due to the decrease in filler dispersion and the appearance of agglomeration phenomenon. At the same time, the distribution density of filler particles in the matrix increased, forming a more compact network structure and leading to a further increase in the hardness of SBR.

### 4.5. Electrical Properties

**Figure 8:**
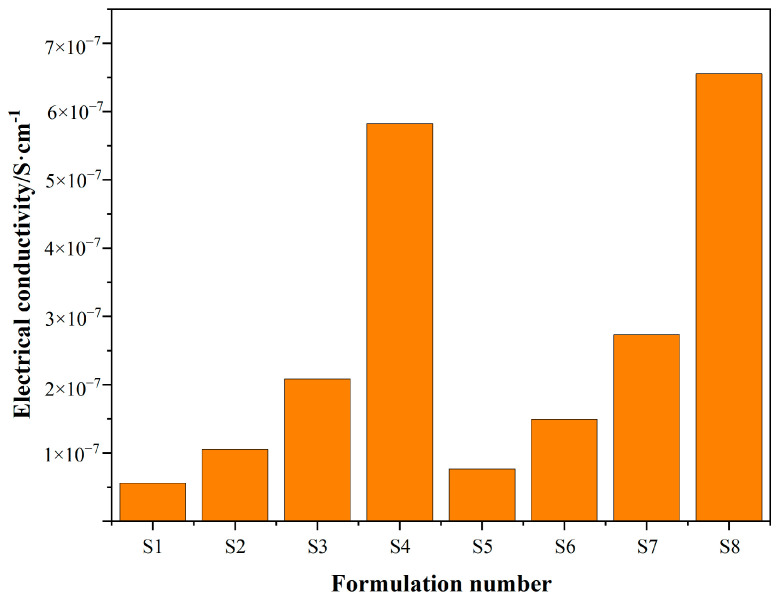
Electrical conductivity of N660 carbon black- and new carbon black-filled SBR.

Figure 8 demonstrates the effect of filling different amounts of N660 carbon black (S1-S4) and new carbon black (S5-S8), respectively, in a 100 phr SBR matrix on the electrical conductivity of vulcanized rubber. The conductivity of both carbon blacks increases with the increase in the filling level. In the N660 carbon black-filled formulation, the conductivity is gradually improved with the increase in the filling amount. Among them, S1 has the lowest conductivity of about 5.58485 × 10^−8^ S/cm, and S4 has the highest conductivity of about 5.82370 × 10^−7^ S/cm. In the new carbon black-filled formulation, the conductivity also increases with the increase in the filling amount. S5 has a conductivity of 7.64468 × 10^−8^ S/cm, which is about 4% higher than that of the N660 carbon black-filled SBR composite with the same filling amount. The conductivity of S5 was 7.64468 × 10^−8^ S/cm, which was about 4% higher than that of N660 carbon black-filled SBR composites with the same filling amount, and the conductivity of the S8 formula reached 6.55498 × 10^−7^ S/cm, which was about 13% higher than that of N660 carbon black-filled SBR composites with the same filling amount.

Comparative results show that the conductivity of the new carbon black is overall better than that of N660 carbon black at the same filler level, and this difference is more significant at high filler levels. This could be attributed to the microstructure and interfacial properties of the new carbon black, which allows it to form a more efficient conductive network within the composites. This performance advantage highlights the potential of new carbon black for applications in conductive materials [17].

## 5. Conclusions

By analyzing the properties of the new carbon black, the following conclusions were drawn: the new carbon black can partially replace N660 carbon black as a reinforcing filler for SBR. When the filling amount of the new carbon black is 30 phr per 100 phr of SBR, the tensile strength is increased by 12.3% and the tear strength is increased by 9.6% compared with N660 carbon black-filled SBR composites, while the hardness is reduced. The new carbon black helps improve the vulcanization efficiency of SBR, which can shorten the vulcanization time t_90_ of SBR and ensure the uniformity and stability of the vulcanization reaction. In addition, the new carbon black has a stronger conductive reinforcement effect due to its lamellar structure, larger specific surface area, and multi-defect structure. Finally, the new carbon black has economic and environmental advantages—its price is only two-thirds of N660 carbon black and environmental protection is higher—to meet the green low-carbon production requirements.

## Figures and Tables

**Table 1:** Variable.

Component	Sample Number							
	S1	S2	S3	S4	S5	S6	S7	S8
New carbon black	0	0	0	0	20	30	40	50
N660 carbon black	20	30	40	50	0	0	0	0

**Table 2:** Vulcanization properties of N660 carbon black- and new carbon black-filled SBR.

Project	Sample Number							
	S1	S2	S3	S4	S5	S6	S7	S8
M_H_/(dN·m)	16.916	19.506	22.384	23.83	16.123	18.275	19.999	20.75
M_L_/(dN·m)	0.948	1.135	1.304	1.364	1.057	1.397	1.798	2.122
M_H_ − M_L_/(dN·m)	15.968	18.371	21.08	22.466	15.066	16.878	18.201	18.628
t_90_/min	7:18	6:37	6:05	5:39	5:47	6:05	6:10	6:17

**Table 3:** Mechanical properties of N660 carbon black- and new carbon black-filled SBR.

Project	Sample Number							
	S1	S2	S3	S4	S4	S6	S7	S7
Shore A hardness/A	55	59	64	67	54	57	61	64
Tensile strength/MPa	9.7	16.3	21.27	22.43	10.4	18.3	22.2	23.6
Elongation at break/%	214.3	258.7	259.82	232.02	211.8	222.2	279.1	255.4
Tear strength/kN·m^−1^	22.4	33.46	40.02	47.67	24.46	35.53	43.86	50.87

## Data Availability

Data are contained within the article.
